# Changes in Refractive Error During Young Adulthood: The Effects of Longitudinal Screen Time, Ocular Sun Exposure, and Genetic Predisposition

**DOI:** 10.1167/iovs.64.14.28

**Published:** 2023-11-20

**Authors:** Samantha Sze-Yee Lee, Gareth Lingham, Carol A. Wang, Santiago Diaz Torres, Craig E. Pennell, Pirro G. Hysi, Christopher J. Hammond, Puya Gharahkhani, Rosie Clark, Jeremy A. Guggenheim, David A. Mackey

**Affiliations:** 1 Centre for Ophthalmology and Visual Science (incorporating the Lions Eye Institute), the University of Western Australia, Perth, Western Australia, Australia; 2 Centre for Eye Research Ireland, School of Physics, Clinical and Optometric Sciences, Technological University Dublin, Dublin, Ireland; 3 School of Medicine and Public Health, College of Health, Medicine and Wellbeing, The University of Newcastle, Callaghan, New South Wales, Australia; 4 Hunter Medical Research Institute, New Lambton Heights, New South Wales, Australia; 5 QIMR Berghofer Medical Research Institute, Brisbane, Queensland, Australia; 6 Faculty of Medicine, The University of Queensland, Brisbane, Queensland, Australia; 7 John Hunter Hospital, Department of Obstetrics and Gynaecology, Newcastle, New South Wales, Australia; 8 King's College London, Section of Ophthalmology, School of Life Course Sciences, London, United Kingdom; 9 King's College London, Department of Twin Research and Genetic Epidemiology, London, United Kingdom; 10 School of Biomedical Sciences, Queensland University of Technology, Brisbane, Queensland, Australia; 11 School of Optometry & Vision Sciences, Cardiff University, Cardiff, United Kingdom; 12 Centre for Eye Research Australia, Royal Victorian Eye and Ear Hospital, University of Melbourne, East Melbourne, Victoria, Australia; 13 School of Medicine, Menzies Research Institute Tasmania, University of Tasmania, Hobart, Tasmania, Australia

**Keywords:** genetics, myopia, polygenic score (PGS), screen time, the Raine Study, young adults

## Abstract

**Purpose:**

Changes in refractive error during young adulthood is common yet risk factors at this age are largely unexplored. This study explored risk factors for these changes, including gene–environmental interactions.

**Methods:**

Spherical equivalent refraction (SER) and axial length (AL) for 624 community-based adults were measured at 20 (baseline) and 28 years old. Participants were genotyped and their polygenic scores (PGS) for refractive error calculated. Self-reported screen time (computer, television, and mobile devices) from 20 to 28 years old were collected prospectively and longitudinal trajectories were generated. Past sun exposure was quantified using conjunctival ultraviolet autofluorescence (CUVAF) area.

**Results:**

Median change in SER and AL were −0.023 diopters (D)/year (interquartile range [IQR] = −0.062 to –0.008) and +0.01 mm/year (IQR = 0.000 to 0.026), respectively. Sex, baseline myopia, parental myopia, screen time, CUVAF, and PGS were significantly associated with myopic shift. Collectively, these factors accounted for approximately 20% of the variance in refractive error change, with screen time, CUVAF, and PGS each explaining approximately 1% of the variance. Four trajectories for total screen time were found: “consistently low” (*n* = 148), “consistently high” (*n* = 250), “consistently very high” (*n* = 76), and “increasing” (*n* = 150). Myopic shift was faster in those with “consistently high” or “consistently very high” screen time compared to “consistently-low” (*P* ≤ 0.031). For each z-score increase in PGS, changes in SER and AL increased by −0.005 D/year and 0.002 mm/year (*P* ≤ 0.045). Of the three types of screen time, only computer time was associated with myopic shift (*P* ≤ 0.040). There was no two- or three-way interaction effect between PGS, CUVAF, or screen time (*P* ≥ 0.26).

**Conclusions:**

Higher total or computer screen time, less sun exposure, and genetic predisposition are each independently associated with greater myopic shifts during young adulthood. Given that these factors explained only a small amount of the variance, there are likely other factors driving refractive error change during young adulthood.

Continued changes in refractive error during, in particular a myopic shift, during young adulthood has been widely reported.^1^^–^^5^ However, these observations have often been restricted to university students and may thus give the impression that higher education is directly linked to myopigenesis during young adulthood. Our recent study^6^ demonstrated that myopia progression and onset remain common during young adulthood and were unrelated to higher education, and major risk factors of myopic refractive shift were mostly non-modifiable, including parental myopia and female sex.^6^

Risk factors for childhood myopia have been extensively studied, but those during young adulthood have received little attention. With the introduction of smartphones and a shift toward a reliance on technology, there is an increasing focus on digital screen time as a potential risk factor for myopia; however, the findings among children are somewhat mixed.^7^^–^^9^ The current generation of young adults, the “millennial” generation, are a particularly interesting group to study. Millennials are the first generation to grow up with the internet, whereas iPhones became ubiquitous a few years after its release in 2008,^10^ when this generation was in their early teens to mid-twenties.

In addition, the genetic contribution to myopic shift during young adulthood and how it may interact with environmental factors remains unclear. This study explored potential risk factors for myopic shift during early adulthood, including screen time and a polygenic score (PGS) for refractive error, as well as gene–environment interaction effects. Importantly, rather than relying on cross-sectional data, this study modeled the longitudinal patterns of screen and outdoor time during early adulthood, allowing us to investigate how changes in these measures contribute to changes in refractive error including myopia shifts.

## Methods

This study comprises a subset of the Raine Study's Gen2 participants, a cohort of 2868 born in 1989 to 1992 at the King Edward Memorial Hospital in Perth, Western Australia. These participants have been followed since prenatally with a series of health examinations and questionnaires.^11^ At the 20-year visit (2010–2012), participants underwent a baseline eye examination and a follow-up was conducted in 2018 to 2020. All assessments in the Raine Study complied with the Declaration of Helsinki and have been approved by the University of Western Australia Human Research Ethics Committee. All participants provided written informed consent following a full explanation of the study prior to each assessment.

### Eye Examination

The 20-^12^ and 28-year^6^ eye examination protocols have been described previously. Spherical equivalent refractive error (SER) was measured using an autorefractor (Nidek ARK-510A; NIDEK) at least 20 minutes after instillation of 1% tropicamide. Axial length (AL) was measured using an IOLMaster V5 (Carl Zeiss Meditec AG). Conjunctival ultraviolet autofluorescence (CUVAF) photography was conducted with custom camera and lenses and the CUVAF area measured,^13^ with larger areas indicating more sun exposure. Ocular sun exposure between the 20- and 28-year eye examinations was quantified as the average annual rate of change in CUVAF area between the 2 visits. Participants who had refractive surgery between the 20- and 28-year examinations were asked to provide their refractive error prescription prior to surgery via self-report or from their optometrist. The presurgical prescription was added to the refractive error measured at the 28-year follow-up and included in the analysis.^6^ Individuals with keratoconus, refractive surgery with unknown presurgical prescription, or orthokeratology lens wear were excluded from the SER analysis, but included in the AL analysis. A person was defined as having myopia if they have a SER of ≤−0.50 diopters (D) in either eye.^14^

### Questionnaires

Information on parental myopia, screen time, and time outdoors, were collected prospectively using self-administered questionnaires at 20, 22, 27, and 28 years. Based on the questionnaires, we identified three major types of screen time: computers (desktops and laptops), television (including console games), and handheld mobile devices (smartphones and tablets). The detailed method of estimating the daily average screen and outdoor time based on the participant-reported information are detailed in the Supplementary Notes S1.

### Trajectory Modeling

Trajectory modeling is an approach to identify subgroups (i.e. trajectory group) within a population based on patterns of observed variables (e.g. self-reported screen time), and can be applied in longitudinal datasets. To achieve this, latent class mixed modeling (LCMM) was conducted using the “lcmm” package in R version 4.0.2 (The R Foundation for Statistical Computing Platform, Vienna, Austria).^15^

For each screen or outdoor time variable, a series of LCMMs were generated with 1 to 6 latent classes (trajectory groups)^16^ with 100 iterations, and random intercepts and slope for year of visit (20-, 22-, 27-, and 28-year). The optimal number of trajectory groups for each variable was determined based on: (1) the minimum Bayes and Akaike's information criteria values; (2) ≥ 5% of the study population in each trajectory group; (3) distinct and meaningful patterns in the modelled trajectories; and (4) an average posterior probability (probability of correct classification) of 70%.^16^^–^^19^ Participants were assigned to the trajectory group to which they have the highest probability of membership.

### Genotyping and Quality Control

Whole blood samples were collected at 14 and 17 years. Samples from 1592 participants were analyzed in 2010 using an Infinium HD Human 660W-Quad BeadChip Array and those from an additional 310 participants were analyzed in 2013 using an Infinium OmniExpress-24 BeadChip Array. For quality control, data with missingness per single nucleotide polymorphism (SNP) or per person > 0.05, and a Hardy-Weinberg equilibrium *P* value < 10^−6^ or a minor allele frequency < 0.01 were excluded. These were then imputed against the Haplotype Reference Consortium panel^20^ using the Michigan Imputation Server.^21^ Imputed SNPs with an imputation quality score > 0.3 were included in the analysis. Principal component analysis was conducted prior to imputation using the 1000 Genomes (Genomes Project Consortium 2015) as a reference.

### Polygenic Scores

The PGS for refractive error was calculated for each participant using PLINK 2, based on the PGS developed by Clark et al.,^22^ available at https://figshare.com/articles/dataset/Polygenic_score_PGS_for_Refractive_Error_eBioMedicine_/22294390. Briefly, the PGS was generated from the summary statistics of the genome-wide association study (GWAS) for average spherical equivalent for three European cohorts: the United Kingdom (UK) Biobank participants (*n* = 101,523), the Consortium for Refractive Error and Myopia (*n* = 42,060), and the Genetic Epidemiology Research on Adult Health and Aging (GERA; *n* = 34,998), as well as the GWAS of 290,188 non-overlapping UK Biobank participants with refractive error inferred from their age-of-onset of spectacle wear. A PGS comprising approximately 770,000 SNPs was generated and standardized to a z-score with a mean of 0 and a standard deviation of 1, for interpretation purposes.

### Statistical Analysis

All analyses were conducted on R and *P* < 0.05 was taken as statistical significance. Primary outcomes were the annual rates of change in SER and in AL. These were calculated as the difference in values between 28- and 20-year data, then dividing by the amount of time (in years) between the 2 visits, and averaged between both eyes. Linear regressions were used to explore the associations of each outcome measure with its baseline value (SER or AL), sex, CUVAF area, parental myopia, total screen time, and PGS, as well as the interaction effects between these variables. Despite the non-normal distribution of the outcome measures, linear regression is an appropriate method of analysis given our large sample size;^23^^,^^24^ it additionally allows us to obtain an R^2^ value for the explanatory measures of interest. We then repeated the analyses substituting total screen time with computer, television, or mobile device time to explore each of their associations with refractive error change. Additionally, models were regenerated substituting change in CUVAF area with trajectory of self-reported time outdoors, as well as replacing baseline value (SER or AL) with myopia status as baseline. Given the potential for a high correlation between some explanatory parameters, those with variance inflation factor > 5, with the exception of interaction terms, were removed from the model.

Linear regression models additionally controlled for baseline age, posterior probability of trajectory group,^25^ genotyping array, and/or the first 10 genetic ancestry principal components, where applicable. From the linear regression models, the R package “boot” was used to calculate the variance (R^2^) in refractive error changes explained by each independent measure of interest, as well is the 95% confidence interval (CI). These were calculated as the difference in R^2^ between the full model and a model without the explanatory variable of interest. Additionally, we interrogated the predictive performance of the PGS on refractive error measured at 20 and 28 years.

## Results

### Study Sample

**Figure 1.**
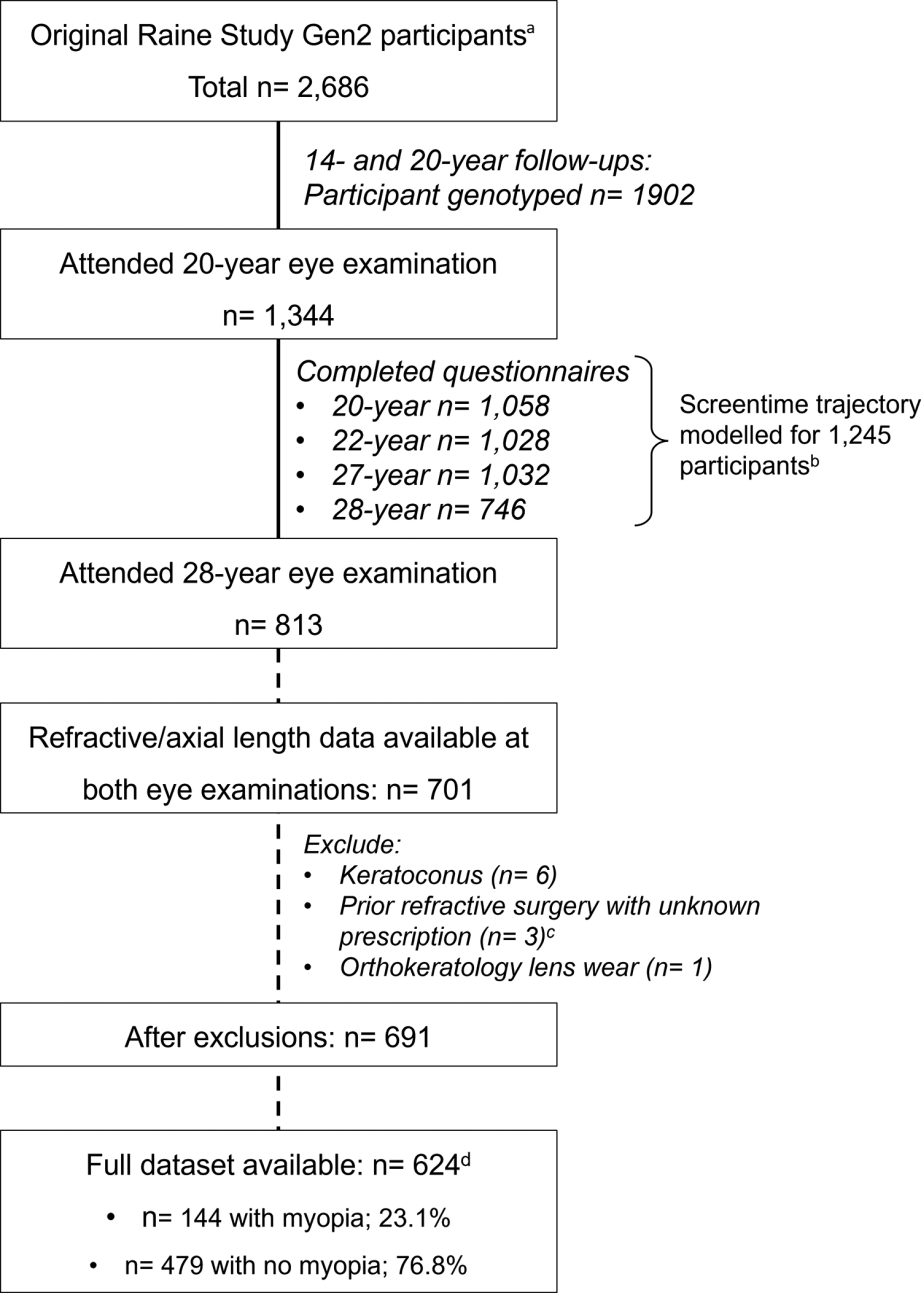
Sample size at each visit. (**a**) Participants' numbers shown at each visit may include those who missed a preceding follow-up. (**b**) Each participant only requires a minimum of two longitudinal data points for a trajectory to be modeled, there are thus more participants with trajectory modeled than in each follow-up. (**c**) Participants with known prescription just prior to laser refractive surgery were included in analyses. (**d**) Participants with available data for spherical equivalent or axial length, polygenic score, screentime, and time spent outdoors.

Figure 1 shows the number of participants who completed the eye examinations and questionnaires at each visit. A total of 624 participants had full SER or AL data at both eye examinations and genetic or screen time trajectory data, including 144 (23.1%) who had myopia at baseline and 4 who had laser refractive surgery. Supplementary Table S1 shows the participants demographics and refractive measures at the 2 eye examinations.

**Figure 2.**
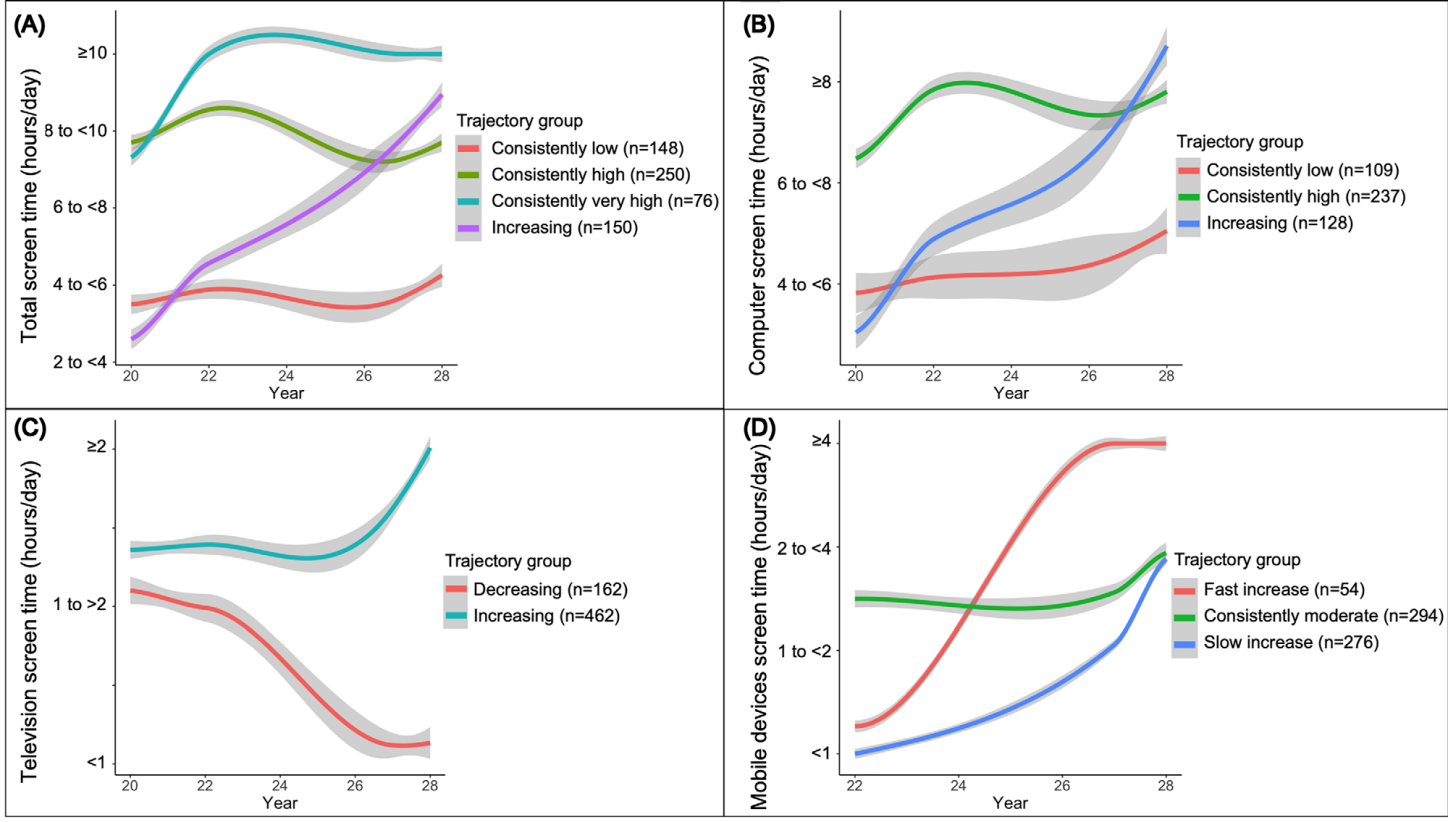
Trajectory plots for (**A**) total screen time; (**B**) computer; and (**C**) television watching between 20 and 28 years old, and (**D**) mobile device use between 22 and 28 years old, as well as the number of participants in each trajectory group. Shaded areas represent the 95% confidence interval of the mean. Note the difference in y-axis scale.

For total screen time, four trajectory groups were identified; participants were classified as having “consistently low,, “consistently high,, “consistently very high,, or “increasing” screen time. Screen time trajectories for computer (3 groups), television (2 groups), and mobile devices (3 groups) and the number of participants in each group are shown in Figure 2.

**Figure 3.**
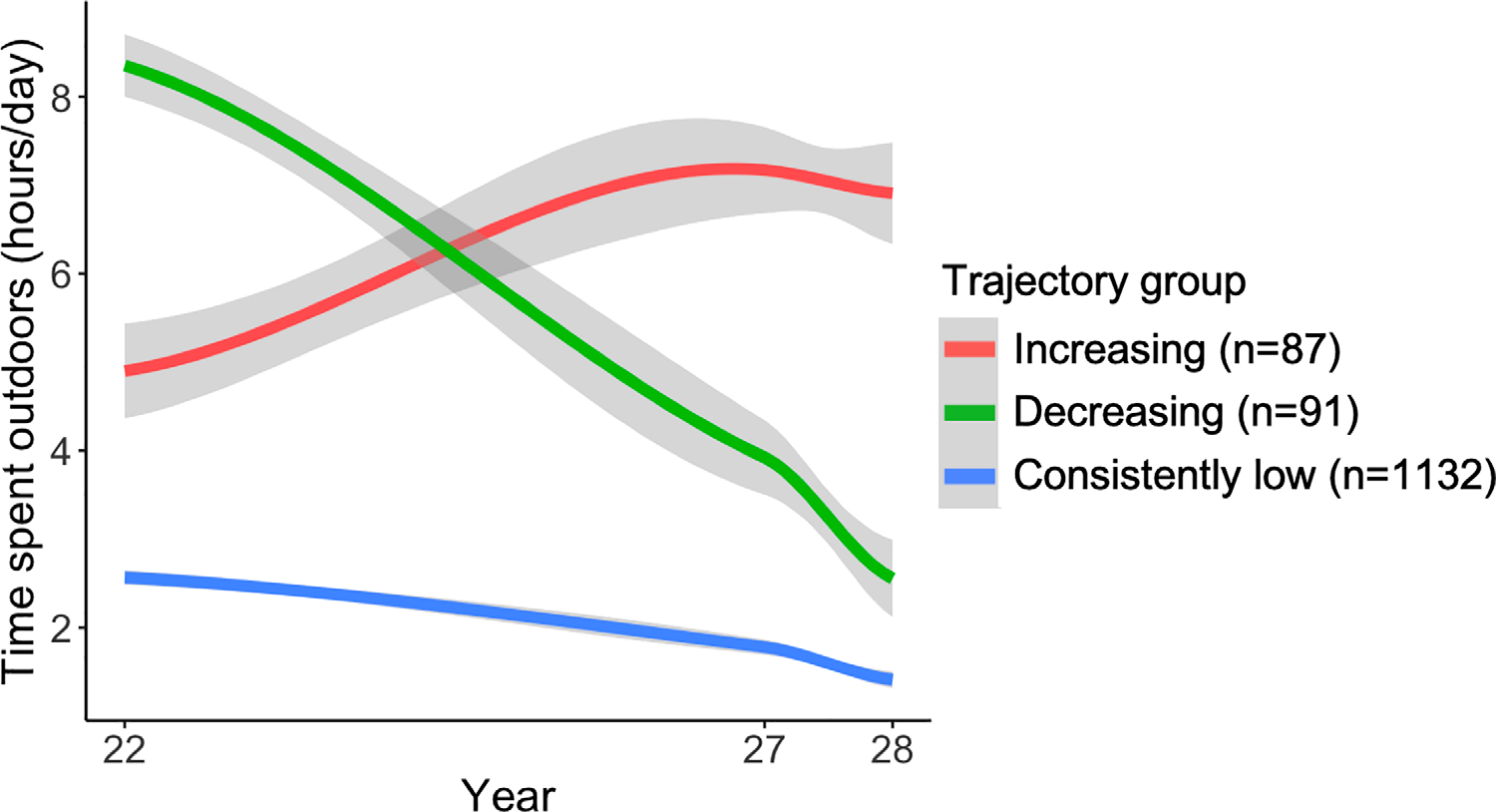
Trajectory plot for self-reported time spent outdoors between 22 and 28 years old and the number of participants in each trajectory group. Shaded areas represent the 95% confidence interval.

For self-reported time spent outdoors, three trajectory groups were identified: “increasing,” “decreasing,” and “consistently low.” As shown in Figure 3, the vast majority of participants had “consistently low” time spent outdoors, averaging around 2 to 3 hours per day at 22 years to approximately 1 hour/day at 28 years.

### Myopic Shift in Refractive Error

Faster rates of change in SER and AL were both associated with female sex, increased screen time, less CUVAF area, and higher PGS (Table 1). A more negative SER at baseline was also associated with a faster longitudinal decrease in SER, whereas longer baseline AL was associated with faster axial elongation, suggesting that a more myopic refractive error at baseline is linked with faster myopia progression. Parental myopia was associated with faster axial elongation, but not SER change. None of the explanatory variables in the following multivariable models had a variance inflation factor > 5, and thus all remained in the models. There were no other interaction effects among sex, baseline values, parental myopia, CUVAF, total screen time, or PGS on change in SER or AL (all *P* ≤ 0.26). The multivariable model shown in Table 1 explained 19.4% (95% CI = 12.7–26.7%) and 20.0% (95% CI = 13.4–27.4) of the variance in change in SER and AL, respectively.

**Table 1.** Effect of Each Explanatory Variable on Longitudinal Change in Spherical Equivalent and Axial Length^*^

	Univariable Analysis		Multivariable Analysis^†^	
	Estimate (95% CI)	*P* Value	Estimate (95% CI)	*P* Value
***Change in spherical equivalent (D/y*** ***)***				
Female sex (ref = male)	−0.037 (−0.052 to −0.022)	<0.001	−0.035 (−0.050 to −0.020)	<0.001
Baseline spherical equivalent (D)	0.021 (0.015 to 0.027)	<0.001	0.019 (0.013 to 0.025)	<0.001
CUVAF area (z-score/year)	0.003 (−0.001 to 0.006)	0.20	0.004 (0.001 to 0.008)	0.029
Number of parents with myopia (per parent)	−0.018 (−0.030 to −0.006)	0.003	−0.005 (−0.017 to −0.007)	0.41
*Screen time trajectory (ref = consistently low)* ^‡^				
•Consistently high	−0.018 (−0.038 to 0.002)	0.07	−0.011 (−0.030 to 0.009)	0.29
•Consistent very high	−0.050 (−0.077 to −0.023)	<0.001	−0.032 (−0.058 to −0.005)	0.019
•Increased from low to high	−0.025 (−0.047 to −0.003)	0.026	−0.022 (−0.043 to 0.001)	0.10
Polygenic score (z-score)	−0.010 (−0.018 to −0.002)	0.014	−0.004 (−0.010 to −0.005)	0.045
***Change in axial length (mm/y*** ***)***				
Female sex (ref = male)	0.011 (0.006 to 0.015)	<0.001	0.013 (0.009 to 0.017)	<0.001
Baseline axial length (mm)	0.009 (0.006 to 0.011)	<0.001	0.008 (0.006 to 0.011)	<0.001
CUVAF (mm/y)	−0.001 (−0.002 to −0.000)	0.009	−0.002 (−0.003 to −0.001)	<0.001
Number of parents with myopia (per parent)	0.007 (0.004 to 0.011)	<0.001	0.004 (0.001 to 0.007)	0.013
*Screen time trajectory (ref = consistently low)*				
•Consistently high	0.009 (0.003 to 0.015)	0.002	0.006 (0.001 to 0.012)	0.020
•Consistent very high	0.011 (0.003 to 0.019)	0.005	0.009 (0.001 to 0.016)	0.019
•Increased from low to high	0.006 (−0.001 to 0.012)	0.07	0.004 (−0.001 to 0.010)	0.14
Polygenic score (z-score)	0.005 (0.003 to 0.008)	<0.001	0.002 (0.001 to 0.005)	0.007

* Only main effects are presented in this table; there was no significant interaction effects; analyzed using linear regression, where applicable, controlled for genetic ancestry (principal components = 1–10), genotyping array, and probability of correct trajectory group classification.

† The multivariable model includes all variables in the current table, additionally controlled for baseline age, genetic ancestry (principal components = 1–10), genotyping array, and probability of correct trajectory group classification.

‡ Total screen time between 20 and 28 years, includes computer, television, and mobile devices.

CI = confidence interval; CUVAF = conjunctival ultraviolet autofluorescence.

When we replaced the baseline value with the presence of myopia at baseline in the models, there were baseline myopia × sex interaction effects on both the SER (*P* = 0.008) and AL (*P* = 0.023). This was such that SER decrease and axial elongation were at approximately three times faster in women with baseline myopia compared to those without (Supplementary Table S2). On the other hand, men with myopia at baseline only had slightly faster axial elongation than those without, by −0.01 D/year (*P* = 0.020), but the difference in rate of SER change did not reach statistical significance (*P* = 0.07).

### Effect of Screen Time

As shown in Table 1, compared to those with “consistently low” total screen time, participants in the “consistently very high” group had faster SER decrease. However, the difference between “increasing” and “consistently low” was not statistically significant in the multivariable analysis.

Faster axial elongation was noted in participants with “consistently high” or “consistently very high” screen time compared to “consistently low” (see Table 1). However, the variance in change in SER and AL explained by total screen time was low, only 1.2% (95% CI = 0.0–3.4) and 0.1% (95% CI = 0.0–2.3), respectively.

Of the three types of screen time, only computer time was significantly associated with a myopic shift in refractive error (Table 2). After accounting for sex, baseline value, CUVAF area, parental myopia, and PGS, participants with “consistently high” or “increasing” computer time had faster SER decrease and axial elongation, compared to those with “consistently low” computer time.

**Table 2.** Effect of Each Type of Screen Time Trajectory on Myopia Progression^*^

	Univariable		Multivariable^‡^	
	Estimate (95% CI)	*P* Value	Estimate (95% CI)	*P* Value
***Change in spherical equivalent (D/y*** ***)***				
*Computer time (ref = consistently low)* ^‡^				
•Consistently high	−0.015 (−0.037 to 0.007)	0.18	−0.015 (−0.030 to −0.000)	0.044
•Increased from low to high	−0.026 (−0.051 to −0.000)	0.048	−0.026 (−0.052 to −0.001)	0.042
Television watching (ref = decreasing)^‡^				
•Increasing	0.004 (−0.110 to 0.017)	0.15	−0.007 (−0.013 to 0.006)	0.50
*Use of mobile devices (ref = slow increase)* ^§^				
•Consistently moderate	−0.003 (−0.032 to 0.026)	0.82	−0.005 (−0.027 to 0.018)	0.68
•Fast increase from low to high	0.007 (−0.022 to 0.036)	0.64	−0.002 (−0.021 to 0.024)	0.89
***Change in axial length (mm/y*** ***)***				
*Computer time (ref = consistently low)* ^‡^				
•Consistently high	0.009 (0.003 to 0.015)	0.005	0.005 (0.000 to 0.011)	0.042
•Increased from low to high	0.010 (0.003 to 0.017)	0.007	0.008 (0.002 to 0.015)	0.012
*Television watching (ref = decreasing)* ^‡^				
•Increasing	0.002 (−0.004 to 0.008)	0.48	0.001 (−0.004 to 0.007)	0.69
*Use of mobile devices (ref = slow increase)* ^§^				
•Consistently moderate	0.004 (−0.004 to 0.012)	0.36	0.003 (−0.005 to 0.010)	0.47
•Fast increase from low to high	0.002 (−0.006 to 0.010)	0.67	0.000 (−0.007 to 0.008)	0.94

* Each type of screen time was analyzed in separate linear regression models, controlled for probability of correct trajectory group classification.

‡ The multivariable model corrected for sex, baseline values, baseline age, parental myopia, conjunctival ultraviolet autofluorescence area, polygenic score for refractive error, genetic ancestry (principal components = 1–10), genotyping array, and probability of correct trajectory group classification.

‡ Trajectory between 20 and 28 years.

§ Trajectory between 22 and 28 years.

CI = confidence interval.

### Effect of Time Outdoors

Greater increase in CUVAF area was associated with slower change in SER and AL (see Table 1), although it only explained 0.7% (95% CI = 0.0–2.8) and 1.3% (95% CI = 0.1–3.5) of the variance in SER and AL change, respectively. However, trajectory of self-reported time outdoors, which replaced the CUVAF area in separate models, was not associated with change in SER or in AL (*P* ≥ 0.18).

### Polygenic Score

PGS accounted for 7.4% (95% CI = 4.0–11.8) and 4.0% (95% CI = 1.6–7.2) of the variance in SER and AL at the 20-year visit, respectively. This is similar to the R^2^ of 7.4% (95% CI = 4.1–11.8) for SER and 4.8% (95% CI = 2.0–8.4) for AL observed at the 28-year visit. For the 8-year change, PGS explained 0.9% (95% CI = 0.0–2.0) and 1.0% (95% CI = 0.7–3.1) of the variance in SER and AL, respectively.

Given that the current PGS has been shown to have better predictive performance in European than non-European individuals, we repeated these analyses in only the European participants (*n* = 568). The R^2^ increased by an additional 1.0% to 1.6% in the 20- and 28-year cross-sectional measures when only European participants were included, but the R^2^ for longitudinal change remained the same (Table 3).

**Table 3.** Effect of Polygenic Score (PGS) in European Participants Only (*n* = 568)

	Effect of PGS (95% CI; per z-Score)	PGS R^2^ (95% CI)	*P* Value^*^
**20-y eye examination**			
Spherical equivalent (D)	−0.45 (−0.57 to −0.34)	9.0% (5.2 to 14.0)	<0.001
Axial length (mm)	0.21 (0.14 to 0.28)	5.1% (2.4 to 8.8)	<0.001
**28-y eye examination**			
Spherical equivalent (D)	−0.53 (−0.66 to −0.40)	9.2% (5.2 to 14.1)	<0.001
Axial length (mm)	0.25 (0.17 to 0.32)	5.8% (2.7 to 9.8)	<0.001
**8-y change**			
Spherical equivalent (D/y)	−0.005 (−0.011 to 0.001)	0.0% (−0.0 to 2.0)	0.054
Axial length (mm/y)	0.003 (0.001 to 0.005)	0.8% (0.0 to 2.)	0.010

* Analyzed using linear regression, corrected for sex, baseline values, parental myopia, conjunctival ultraviolet autofluorescence, genetic ancestry (principal components = 1–10), genotyping array, screen time trajectories, probability of correct screen time trajectory group classification, and, for the 8-year change, baseline age.

CI = confidence interval; PGS = polygenic score.

## Discussion

Despite the common occurrence of changes in refractive error, including myopia progression, during young adulthood,^26^ our understanding of the risk factors and etiology of such changes in this age group remains limited. This is an important area to explore as it would allow clinicians to determine management of individuals who are at risk of progressing to high myopia during the third decade of life. For example, we previously reported a case^27^ of a study participant (who was also part of the current study cohort) whose myopia progressed by 5.0 D between 20 and 28 years of age (from approximately −4 D to −9 D in the worse eye), whereas another article described an individual whose myopia progressed from −0.50 D in their early 20s to −11 D in their mid- to late 30s, later developing myopic macular degeneration.^28^ In a previous manuscript,^6^ we reported a 0.7% incidence of high myopia between 20 and 28 years old. Although this is a relatively low rate, it may be wise to anticipate an increase in incident high myopia during young adulthood over the next few decades, paralleling the worldwide trend in childhood myopia epidemic.

Interestingly, women with myopia at baseline had markedly faster SER decrease and axial elongation than those without, whereas the difference between these subgroups in men were smaller. The sex difference could be related to lifestyle factors, but given that we have accounted for sun exposure and screen time, these are unlikely to mediate the sex–baseline myopia relationship. Another possible reason for this interaction effect could be related to biological factors, such as hormonal changes or ocular trait differences between sexes. For example, the central choroid has been reported to be thicker in men than women,^29^^–^^31^ although this has been disputed.^32^^,^^33^ In the current cohort, we had previously found that the choroids are approximately 17 µm thicker in men than in women at the 20-year examination (baseline), after accounting for axial length and transverse magnification.^34^ We later reported that thicker baseline choroids could be protective against axial elongation, even after controlling for baseline refractive error.^35^ Given that women, especially those with baseline myopia, have thinner choroids, we suspect that sex differences in choroidal thickness, or other ocular traits, could mediate the sex–baseline myopia interaction effect on myopic shift. However, at this stage, this is purely speculative and further studies are required to investigate this.

Increased screen time or near work has long been believed to be causal of myopia. However, because less time spent outdoors is a major risk factor for myopia,^36^ it remains unclear whether the effects of increased screen time or near work are independent of time outdoors.^7^^–^^9^^,^^37^ A limitation of most studies assessing screen time has been that the data are often cross-sectional in nature, usually collected around the time the refractive error was measured. Screen time data collected at the time of eye examination may not reflect screen time in the preceding years when myopia is developing or progressing.

As demonstrated in the current study using trajectory modeling, total screen time dramatically increased between 20 and 28 years in about one-quarter of adults, whereas more than one-third of young adults had large increases in computer time during the same period.

Importantly, of the three types of screen time, only computer time was significantly associated with faster myopic shift. The effect of “increasing” computer time on the myopic shift in refractive error is likely related to a shift toward a dependency on digital technology. An “increasing” trajectory of computer time does not necessarily suggest that total near work is increasing, and may instead reflect a replacement of traditional forms of near work, such as pen-and-paper reading or writing, with computer usage. Unfortunately, longitudinal information on non-digital near work is not available in the current cohort to verify this.

Interestingly, an association between “increasing” total screen time and change in refractive error failed to reach statistical significance in the multivariable model. An “increasing” total screen time may be driven by mobile devices or television screen time, neither of which are linked with a myopic shift in refractive error, at least in the current cohort. The lack of association between television watching and refractive error change in our sample of young adults is in concordance with findings in children^38^^–^^40^ or university students.^41^ In contrast to our observation, a meta-analysis^7^ recently reported a significant link between mobile device and myopia progression. However, the authors^7^ acknowledged that many studies failed to account for potential confounders and it remains unclear if use of smart or mobile devices is an independent myopia risk factor. In a cohort of 1884 adolescents, Toh et al.^42^ even reported a small protective effect of smartphone use. Unlike computers and televisions, smartphones and tablets are extremely portable and can easily be used outdoors. Built-in features, such as the camera and global positioning system, are likely to also increase the use of these devices outdoors. We additionally propose that interacting with the small screens of hand-held smart devices potentially induces peripheral myopic defocus during use, a stimulus known to prevent axial eye growth. Further experimental studies are required to test this theory which, if proven, will potentially have a significant impact on the screen habits of young adults and children.

We considered the possibility that increasing sun exposure could offset the detrimental effect of screen time. Although there is some protective effect of ocular sun exposure, as quantified by the increase in the CUVAF area, there was no interaction effect with screen time.

The variances in SER and AL explained by the PGS were essentially identical between the 20- and 28-year visits. Additionally, the R^2^ of 7.4% for SER at both follow-ups in our young adult sample was remarkably close to the 6.9% previously found in an older population in the same state of Western Australia.^22^ These values are much lower than the 19% and 15% reported in 2 independent UK population-based cohorts, using the same PGS.^22^ The lower R^2^ found in the Australian cohorts may be attributed to at least two reasons. First, the PGS has a poorer predictive ability in individuals of non-European ancestry, as shown in our previous study.^22^ Indeed, after excluding participants of non-European descent, which made up approximately 9% of the current study sample, the variance explained by PGS for SER and AL in the Raine Study increased to 9% and 5%. Second, there is likely to be a gene–environment correlation effect, where people with similar phenotypic features tend to migrate to the same place of residence. Controlling for geographic regions in statistical models can decrease the heritability and genetic correlations of certain non-eye phenotypic features.^43^ Environmental differences between the UK and Australia, such as amount of sunlight and UV levels may have also contributed to the differences in variance explained by the PGS between regions.

We further found that PGS accounted for no more than 1% of the variance in 8-year change in refractive error, and thus the current PGS is not suitable for predicting refractive error progression during young adulthood. The PGS was derived based on refractive error, and may therefore be more suited to describing measured refractive error rather than longitudinal change, especially in young adulthood where the range of change in refractive error is small. This also suggests that other factors may play a more important role in refractive error changes during young adulthood. For example, as found in the current study, having consistently high screen time or less sun exposure is linked to faster decrease in spherical equivalent and axial elongation. However, the R^2^ values for screen time and CUVAF were also very low. Thus, genetic variants not included in our up-to-date PGS that have stronger associations with refractive error change during young adulthood may yet to be discovered, or there may be other unexplored environmental factors for myopia progression at this age. This highlights our poor understanding of factors associated with refractive error change in this age group and suggests the need for future studies to explore other genetic and environmental drivers, which will inform potential preventive measures such as reducing screen time during young adulthood.

A major strength of the current study is its relatively large community-based cohort of young adults that is generally representative of the Western Australia population. Even though the participation rate of the Raine Study Gen2 cohort has dwindled since their enrollment more than 30 years ago, differences in socioeconomic factors between the study participants and Western Australia residents of the same age were all less than 10%, at least up to the 22-year follow-up.^44^ The lower than expected turn out for the 28-year eye examination was partly due to the coronavirus disease 2019 (COVID-19) pandemic, which forced data collection to cease prematurely in 2020. It should also be noted that the attrition rate in any longitudinal study is expected to increase with longer follow-ups and more markedly in younger individuals. For example, the Australian Longitudinal Study on Women's Health had a 32% attrition rate among young (18–23 years old) women in just 4 years, compared to only 10% to 16% in women 45 years and older.^45^ As outlined by Bullimore et al.,^26^ working-age adults are a challenging demographic group to observe longitudinally given that this age tends to be a busy time in life due to work and personal commitments, and may not prioritize participation in research or their eye health given that they still generally have good vision. Thus, longitudinal studies in young adults, whether observational or interventional, will need to plan for a potentially high attrition rate.

Another strength of the study was the use of CUVAF to quantify ocular sun exposure, rather than solely relying on self-reported data of the time outdoors. However, a limitation of CUVAF is that its area tends to decrease with age and sunglass wear,^13^ and we may have therefore underestimated ocular sun exposure in participants who practice sun safe habits. CUVAF measures also do not provide a quantitative time duration, which is more useful for providing advice on amount of time to spend outdoors. Nonetheless, using CUVAF may be advantageous over self-reported outdoor time given the former's objective nature. As with most self-reported data, we were unable to verify the accuracy of our screen and outdoor time data, which are vulnerable to recall biases. Nonetheless, the prospective nature of the questionnaires makes it less prone to such errors, as compared to retrospectively collected data. Collection of this information over a longitudinal period also allowed us to model trajectories, rather than relying on cross-sectional data which do not reflect the myopia-progressing years. These data allowed us to separately analyze the effects of the different types of screen time. Another limitation was the lack of longitudinal data on non-digital near work, which would enable a better understanding of the interplay among near work, screen, and outdoor time on refractive error change.

Although computer time and ocular sun exposure are the only modifiable risk factors that we have found for a faster myopic shift at this age, these factors each explained approximately 1% of the variance in the change in refractive error. Thus, there likely remain other unexplored risk factors for myopia progressing during young adulthood. Given the possibility of rapid myopia progression at this age, it is critical to uncover these other risk factors to inform on the appropriateness of the currently available types of myopia control in this demographic group.

## Supplementary Material

Supplement 1
