# Supplementary material for: Changes in Refractive Error During Young Adulthood: The Effects of Longitudinal Screen Time, Ocular Sun Exposure, and Genetic Predisposition
Source: Invest Ophthalmol Vis Sci. 2023 Nov 20;64(14):28. doi: 10.1167/iovs.64.14.28 (PMC10668617; doi:10.1167/iovs.64.14.28)
Supplement: Supplement 1 [file iovs-64-14-28_s001.pdf]

Supplementary notes 1: Detailed method of estimating average daily screen time and time spent outdoors

a) Screen time

For screen time, at the 20-year baseline visit, participants were asked how many hours they spent each day doing each of the following activities: watching television, playing electronic games not on a computer, and using a computer for work/study/playing games/internet socialising/internet surfing. These questions were again asked at the 22-year visit, in addition to how much time they spent each day using a touch screen tablet or smartphone. For each of these items, participants provided their responses as "not at all", "less than 1 hour", "1–2 hours", "2–4 hours", or "more than 4 hours".

At the 27- and 28-year visits, participants were asked how much time they spent performing the following activities: watching television, using a desktop computer, using a laptop, using a tablet, using a mobile phone, and playing active or non-active games on an electronic console. Participants provided their responses in total minutes per week.

Based on these questionnaires, 3 major types of screen time was identified: computer (desktops and laptops), television (including console games), and handheld mobile devices (smartphones and tablets).

Given that the data at the first 2 visits were collected as categorical responses, for the purpose of trajectory modelling, all screen time variables were analysed as ordinal outcomes. Additionally, total screen time per day was calculated. To estimate the total screen time at 20- and 22-year, we summed up the median value of all the categorical responses (e.g., 1–2 hours would be 1.5 hours). Total screen time per day was then re-categorised and analysed as an ordinal outcome.

Television and mobile devices screen time were each categorized into 4 groups:

- < 1 hour/day
- 1 to <2 hours/day
- 2 to ≤4 hours/day
- 4+ hours/day

Computer time and total screen time were each categorized into 6 groups:

- <2 hours/day
- 2 to ≤4 hours/day
- 4 to ≤6 hours/day
- 6 to ≤8 hours/day
- 8 to ≤10 hours/day
- 10+ hours/day

b) Time spent outdoors

At the 22-year follow-up, participants were asked to report the amount of time they spent outdoors (in hours and minutes) on an average weekday and weekend. At the 27- and 28-year follow-ups, participants reported the amount of time they spent outdoors (in hours and minutes) on an average weekday and weekend in summer and winter.

The de-seasonalized average time spent outdoors per day at each follow-up was then determined from the sum of the total time spent outdoors over summer (hours/day x 90 days of summer in Perth) and winter (hours/day x 92 days of winter in Perth), divided by 182 days.

Supplementary Table 1. Participant demographic and refractive characteristics

Trait	Value
Female (n)	321 (51.4%)
Ethnicity	
• European	572 (91.7%)
• Other	52 (8.3%)
Years spent in formal education (mean ± SD)	16.6 ± 3.7
Highest education level	
• Secondary (high) school	96 (15.4%)
• Undergraduate degree	219 (35.1%)
• Postgraduate degree	105 (16.8%)
• Other	204 (32.7%)
Time between eye examinations (mean ± SD)	8.2 ± 0.5
Rate of change in spherical equivalent (median)	-0.023D/year (IQR= -0.062 to 0.008)
Rate of change in axial length (median)	0.010 mm/year (IQR= 0.000 to 0.026)
20-year eye examination	
Age (mean ± SD)	20.0 ± 0.40 years
N myopia	144 (23.1%)
Spherical equivalent refraction (median)	-0.125 D (IQR= -0.750 to 0.625)
Axial length (median)	23.48 mm (IQR= 23.02 to 24.05)
28-year eye examination	
Age (mean ± SD)	28.2 ± 0.5 years
N myopia	210 (33.6%)
Spherical equivalent refraction (median)	-0.3125 D (IQR= -1.250 to 0.125)
Axial length (median)	23.6 mm (IQR= 23.1 to 24.2)

IQR= interquartile range; SD= standard deviation

Supplementary Table 2. **Median change in SER and AL according to sex and presence of baseline myopia**

	**No myopia at baseline (n= 479)**	**Myopia at baseline (n= 145)**	**Baseline myopia vs no myopia group difference (Estimate [95%CI])***
Female			
Change in SER (D/year)	-0.023 (IQR= -0.062 to +0.000)	-0.083 (IQR= -0.150 to -0.031)	-0.049 [-0.070 to -0.029]; p< 0.001
Change in AL (mm/year)	+0.010 (IQR= -0.001 to +0.022)	+0.030 (IQR= +0.014 to +0.054)	0.018 [0.010 to 0.025]; p< 0.001
Male			
Change in SER (D/year)	-0.008 (IQR= -0.039 to +0.015)	-0.033 (IQR= -0.100 to +0.006)	-0.007 [-0.042 to 0.028]; p= 0.7
Change in AL (mm/year)	+0.007 (IQR= -0.001 to +0.017)	+0.015 (IQR= +0.001 to +0.035)	0.008 [0.001 to 0.015]; p= 0.021

*Analyzed using linear regression, corrected for conjunctival ultraviolet autofluorescence area, parental myopia, total screen time trajectory, and refractive error polyenic score. AL= axial length; CI= confidence interval; IQR= interquartile range; SER= spherical equivalent refraction
